# Identification and Expression Pattern of *cyp26b1* Gene in Gonad of the Chinese Tongue Sole (*Cynoglossus semilaevis*)

**DOI:** 10.3390/ani12192652

**Published:** 2022-10-02

**Authors:** Zhongkai Cui, Jie Wang, Yingming Yang, Zhangfan Chen, Qian Wang, Jialin Wang, Tingting Zhang, Wenteng Xu, Songlin Chen

**Affiliations:** 1 Laboratory for Marine Fisheries Science and Food Production Processes, Pilot National Laboratory for Marine Science and Technology, Yellow Sea Fisheries Research Institute, Chinese Academy of Fishery Sciences (CAFS), Qingdao 266071, China; 2 College of Fisheries and Life Science, Shanghai Ocean University, Shanghai 201306, China

**Keywords:** Chinese tongue sole, *cyp26b1*, gonad development, sex-differentiation

## Abstract

**Simple Summary:**

In fish, it is obvious that the asynchronous development of the gonads and sexual dimorphism limit the development of aquaculture, so the research into sex-differentiation and gonadal growth is very important. Due to the sexual reversal phenomenon (genetic females becoming phenotypic males), the Chinese tongue sole (*Cynoglossus semilaevis*) is a great model for investigating sex-differentiation. Herein, we report one gene involved in sex-differentiation and gonadal growth of the Chinese Tongue Sole. The gene *cyp26b1* (*cytochrome P450 family 26 subfamily b member 1*) is a metabolizing Retinoic Acid (RA) enzyme. Since it regulates RA to control sex determination and differentiation, *cyp26b1* is considered a critical part of mammals’ ovary-antagonizing and testis-determining downstream passageway of *Sry* (*sex-determining region Y*) and *Sox9* (*sry-* *box transcription factor 9*). In fish, the related research is reported only on the Japanese flounder (*Paralichthys olivaceus*) and zebrafish (*Danio rerio*). In the current investigation, the identification and expression pattern of the *cyp26b1* gene in the Chinese tongue sole suggested that *cyp26b1* might impact sex-differentiation and gonadal development.

**Abstract:**

As an RA-metabolizing enzyme, *cyp26b1* has a substantial impact on RA-signaling pathways. The *cyp26b1* gene from the Chinese tongue sole was cloned and identified in this investigation. The *cyp26b1* ORF was 1536 bp in length and encoded a 512 amino acid protein. A quantitative real-time PCR (qPCR) indicated that the *cyp26b1* expression is no significant sexual dimorphism in the gonads at the 80 days post-hatching (dph) stages. After 4 months post-hatching (mph), the expression of *cyp26b1* showed sexual dimorphism and lower level of expression in the ovaries than in the testes. An in situ hybridization demonstrated that *cyp26b1* mRNA was primarily located in the testis. Interestingly, the *cyp26b1* mRNA probe was also detected in the ovaries. These results suggested that *cyp26b1* participates in the sex-differentiation and gonadal development of the Chinese tongue sole.

## 1. Introduction

Sex determination and differentiation are complex processes that depend on a series and interplay of signaling pathways. The development of a fetal gonad into an ovary or testis is determined by the type of genetic signals it receives. In males, the gene *sry* leads to the activation of the signaling pathway of testis development and the suppression of the pathway of ovarian development [1,2]. Knocking-down *DMRT1* (*doublesex and mab-3-related transcription factor 1*) results in the feminization of the testis in chickens [3]. In contrast to the sex determination studies, the genetic mechanism research of controlling the germ and somatic cells’ sexual fate is also important. Both oocytes and sperm are derived from primordial germ cells (PGCs). The germ cells’ (GC) sex is characterized by the somatic cells that surround them [4]. In *Cyp26b1*-null male mouse embryos, endogenous RA is not metabolized, testis determination and steroidogenesis are destructed, ovotestis is formed, and a feminized reproductive tract can be observed [5]. In the Japanese flounder, the *cyp26b1* mRNA expression is increased and the meiotic initiation is delayed during sex differentiation because of high temperature [6]. This indicates that *cyp26b1* is an essential gene for sex-differentiation.

It has been demonstrated that RA can promote GC differentiation, leading to the activation of *Stra8* (*stimulated by retinoic acid gene 8*) in mice [7]. It is mass produced in both female and male mesonephros and only transferred to the developmental mouse ovaries, where it directs GCs to express the *Stra8* gene and begin meiosis. However, the RA of the developing testis is metabolized by the *Cyp26b1*, such that GCs do not enter meiosis [5].

The balance of RA has a relationship with a determination of sex and is essential for spermatogenesis [8]. Playing a substantial physiological role in RA metabolism and inactivating the RA in the somatic cells, *Cyp26b1* has been known to be a factor promoting male germ cell (GC) differentiation [7]. In mice, ensuring that the blocking of GCs differentiation and RA is degraded, the *Cyp26b1* expression is maintained by *Sox9* and *nr5a1* (*Nuclear receptor subfamily 5 group a member 1*; previously known as *Sf1* (*steroidogenic factor 1*) during the development of the gonads [9]. The *Cyp26b1* works downstream of *Sry*, which masculinizes the embryonic gonads through activating male-specific genes such as *Fgf9* (*fibroblast growth factor 9*) and *Sox9* in the somatic cells [10]. By regulating the RA signaling during the beginning of meiosis of GCs in mice, the *Cyp26b1* is reported to be expressed initially in the fetal ovaries and testes 11.5 days post-coitum (11.5 dpc), and is subsequently down-regulated in females but up-regulated in males after 12.5 dpc [11,12]. In the gonadogenesis of mice, the GCs in males and females enter meiosis during the various developmental stages. The low expression level of *Cyp26b1* stimulates RA to cause GCs in the embryonic ovaries to enter meiosis, while in males its up-regulation causes the RA to deteriorate, protecting the GCs from attempting to enter meiosis in the emerging testes [13,14].

By artificial gynogenesis and traditional karyotype analysis studies, Chinese tongue sole have been found to possess a heterogametic sex system (WZ) and pronounced sexual dimorphism, in which males possess two Z sex chromosomes [15]. The females develop much quicker and gain final physical sizes twice or up to four times that of males [15]. Although chromosomal inheritance is the fundamental sex determinant, around 14 percent of genetic ZW females experience sexual reversion to phenotypic males (pseudo-males) [16]. The Chinese tongue sole has the problem of an asynchronous sexual maturity. It takes about 1 year for male fish to reach sexual maturity and 2 years for female fish. These problems affect the work regarding artificial reproduction, so it is necessary to study the sex determination and differentiation of the Chinese tongue sole. Previous research has revealed that *dmrt1* is a vital male-determining sex gene in the Chinese tongue sole [17]. A comparative investigation of the gonadal DNA methylomes and transcriptome analysis of female, pseudo-male, and normal male fish indicated that epigenetic regulation is necessary for the Chinese tongue sole’s sexual reversal [18]. Moreover, in the Chinese tongue sole, numerous sex-associated genes, for instance, *cyp19a1a* (*cytochrome P450 family 19 subfamily a member 1 a*), *foxl2* (*forkhead box protein L2*), *sox9*, *figla* (*folliculogenesis specific BHLH transcription factor*), and *gsdf* (*gonadal somatic cell-derived factor*), have been identified [19,20,21,22]. However, the mechanisms underlying the determination of sex and gonadal growth are unknown. There are few studies on GCs’ differentiation.

In fish, the related research is reported only on Japanese flounders and zebrafish. Our study may provide new insights into the function of teleost *cyp26b1* in sex differentiation. For our objective, we cloned and identified the CDS of *cyp26b1* gene in the Chinese tongue sole. The qPCR expression of *cyp26b1* was examined in various tissues and different stages of the gonads. Furthermore, in situ hybridization was applied to identify the signal of *cyp26b1* in gonads. The results exhibited sexual dimorphism and suggested the involvement of *cyp26b1* in sex-differentiation as well as the gonadal development of the Chinese tongue sole.

## 2. Materials and Methods

### 2.1. Ethics Approval

All experimental protocols were carried out under the aegis of the Yellow Sea Fisheries Research Institute’s animal care and use committee (YSFRI-2021007). To minimize fishes’ suffering, enormous efforts were undertaken, including employing anesthesia by MS222 (Table 1).

### 2.2. Fish and Sample Collection

Experimental fish were purchased from the High-Tech Experimental Base Haiyang (Haiyang, Shandong Province, China). Applying an established method [23], the phenotypic and genetic sexuality were determined. The amplification was performed using the genomic DNA template extracted from fin sample and the sex-specific marker (Table 2), of which male samples produced only 206 bp band and female samples produced 206 and 218 bp bands. A total of 10 tissues (spleen, liver, intestine, kidney, gill, gonads, brain, heart, muscle, and skin) from one-and-a-half-year-old fish were collected. The collected samples were stored for future use at −80 °C after snap-freezing in liquid nitrogen. Furthermore, the 2 yph gonads were stored in 4 percent (*w*/*v*) paraformaldehyde fixative (PFA) at 4 °C overnight and embedded in paraffin for in situ hybridization analysis. In addition, gonads of 80 dph, 4 mph, 6 mph, 1 yph, 1.5 yph, and 2 yph were collected.

### 2.3. Gene Cloning and Phylogenetic Analysis

The ORF was amplified using specific gene primers (Table 2). The Gel Extraction Kit (QIAGEN, Germany) was used to purify the amplified fragments. The purified product was inserted into the pMD18-T vector (Takara, Japan), conveyed into *E. coli* TOP10, and then sequenced. DNASTAR 7.10 (http://www.dnastar.com/) (accessed on 29 November 2021) was applied for forecasting the encoded amino acid and the Open Reading Frame (ORF). The inspection of the conserved domain was performed using the Simple Modular Architecture Research Tool (SMART) (http://smart.embl-heidelberg.de/) (accessed on 18 December 2021). Homologous sequences of amino acids were searched using the BLAST search on NCBI (http://www.ncbi.nlm.nih.gov/BLAST/) (accessed on 18 December 2021). The multiple sequence alignment was performed using the Clustal W at MEGA 7.0 software. The Maximum Likelihood (ML) method was used to create the phylogenetic tree. Bootstrap values were based on 1000 resampling replicates.

### 2.4. Quantitative Real-Time PCR (qPCR)

The total RNA (800 ng) was extracted following the manufacturer’s protocol for the TRIzol reagent (Invitrogen, Carlsbad, CA, USA). The quality of the RNA was measured by NanoVue Plus (Biochrom, Cambridge, Cambs, UK). The genomic contamination in the total RNA was removed using gDNA Eraser (TaKaRa, Otsu, Japan). The reverse transcription was carried out using the PrimeScript™ RT reagent kit (TaKaRa, Otsu, Japan). The experiment followed the principles of independent triplicate analysis and at least three samples were measured. qPCR reaction was performed with a 7500 ABI fast detection system (Applied Biosystems, Foster City, CA, USA) using Takara Green Premix Ex Taq II (TaKaRa, Otsu, Japan). Following the TaKaRa kit manual, 0.4 μL 50×ROX Dye II, 1.0 μL cDNA (0.1–100 ng), 0.4 μL of every primer (10 μM), 10.0 μL 2×SYBR Premix, and 7.8 μL ddH2O were contained in a volume of 20 μL qPCR reaction system. The qPCR program was as follows: 95 °C for 30 s, 40 cycles of 95 °C for 5 s, and 34 s at 60 °C. According to the standard curve, the number of *beta actin* (*actb*) copies in 12 different tissues of the Chinese tongue sole was calculated from the C_t_ values for all samples [24]. The results indicated that *actb* was very stable and its expression was not significantly different (*p* < 0.05) [24]. Thus, *actb* was selected as an internal reference. Applying the one-way analysis of variance (ANOVA) using SPSS18.0 (IBM, New York, NY, USA), the statistical analysis of the data was conducted while setting *p* < 0.05 as the significance.

### 2.5. In Situ RNA Hybridization (ISH)

To locate *cyp26b1* expression in the GCs, ISH, as previously described, was carried out [16]. The *cyp26b1* digoxigenin (DIG)-labeled RNA probes (443 bp) were amplified with the specific primers (Table 2) and inserted into the pGEM-T vector. The selected positive plasmid was linearized with *Not* I and then transcribed by T7 RNA polymerase. The RNA probes were labeled using DIG-NTP by means of the DIG RNA Labeling Kit (Roche, Mannheim, Germany). The deparaffinized slices were incubated with the RNA probes (0.2 µg/mL) at 50 °C overnight. At room temperature, the slices were blocked for 4 h in confining liquid (10% goat serum, 150 mM NaCl, 100 mM maleic acid, and adjusted to pH 7.5). Then, the slices were incubated with the anti-DIG-antibodies (Roche, Mannheim, Germany) overnight. The signal was produced with nitroblue tetrazolium/5-bromo-4-chloro-3-indolyl phosphate (Roche, Mannheim, Germany). Three samples for each group were used for analysis.

## 3. Results

### 3.1. Cloning and Sequencing of the cyp26b1 Gene

**Figure 1:**
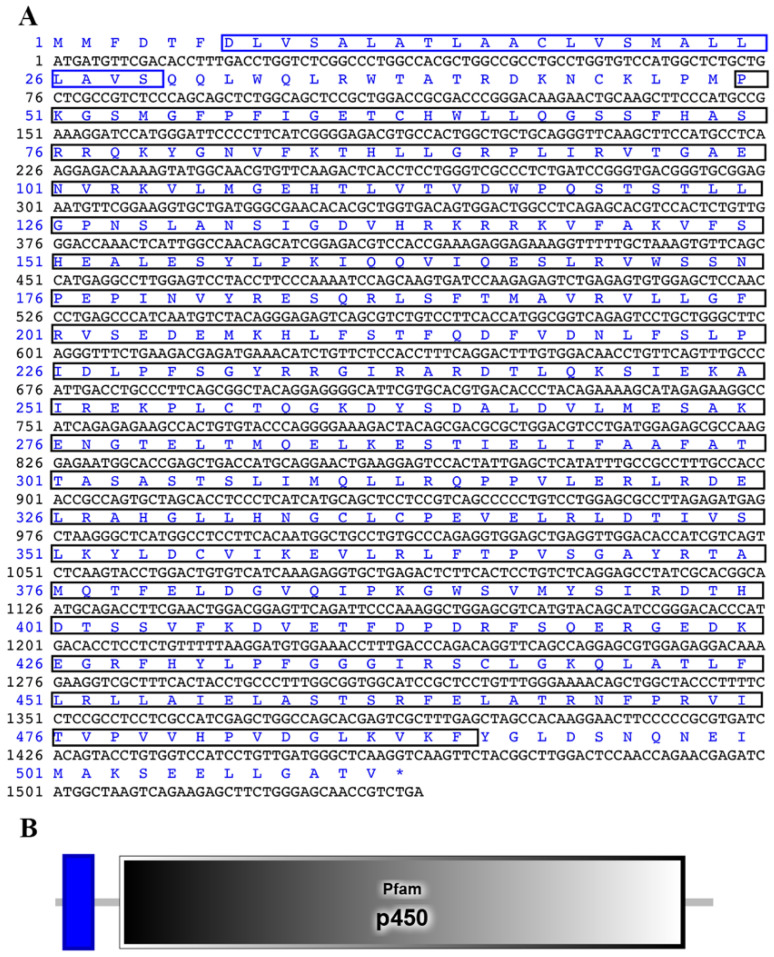
The cyp26b1 (GenBank accession number: XM_008324250.3) ORF and its domains in Chinese tongue sole. (**A**), Nucleotide and the derived pattern of *cyp26b1*. The initiated codon is ATG and stop codon is TGA. The blue boxs represent transmembrane regions and black boxes represent Pfam: p450 domain. (**B**), Domains of *cyp26b1* predicted by SMART program. The transmembrane region is shown with a blue box. The Pfam: p450 domain was marked with gray box.

The ORF of *cyp26b1* was 1536 bp in length (Figure 1A), which encoded a 512 amino acid putative protein with a predicted molecular weight of 57.88 kDa and an isoelectric point (PI) of 8.51. Through searching conserved domains, we determined that the *cyp26b1* gene of the Chinese tongue sole contained the transmembrane region and Pfam: P450 domain (Figure 1).

### 3.2. Phylogenetic Analysis

**Figure 2:**
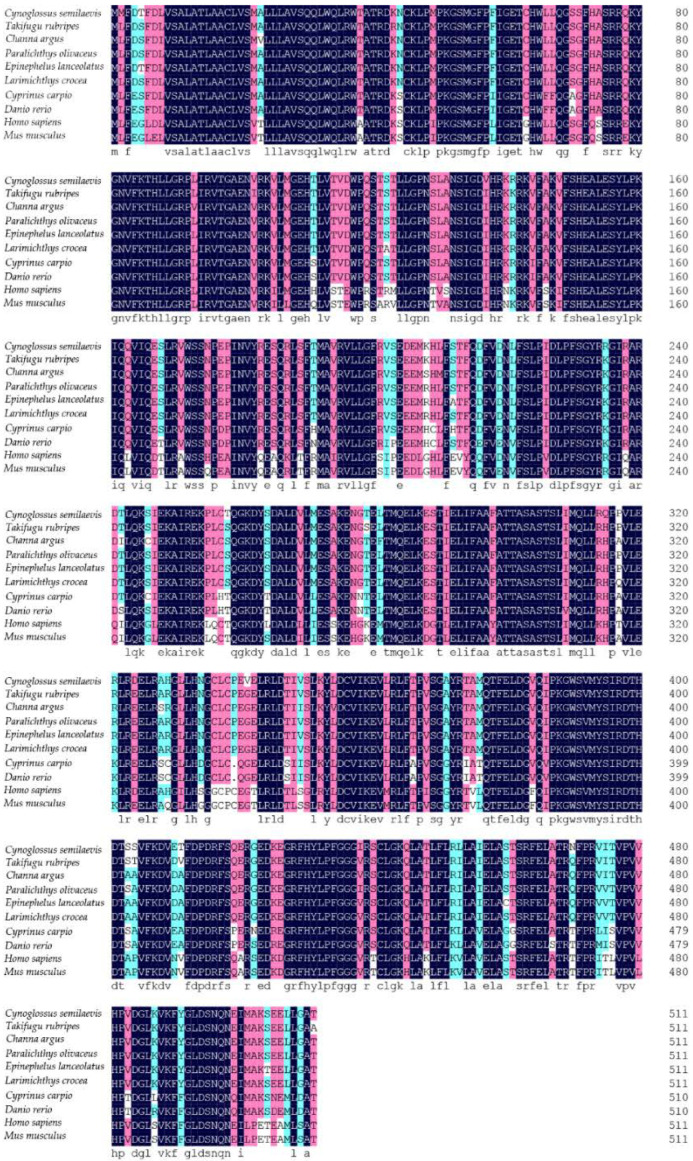
Multiple alignment of the Chinese tongue sole Cyp26b1 protein sequences with other vertebrates. The consensus residues are in black, the residues that are ≥75% identical among the aligned sequences are in pink, and the residues that are ≥50% identical among the aligned sequences are in blue.

**Figure 3:**
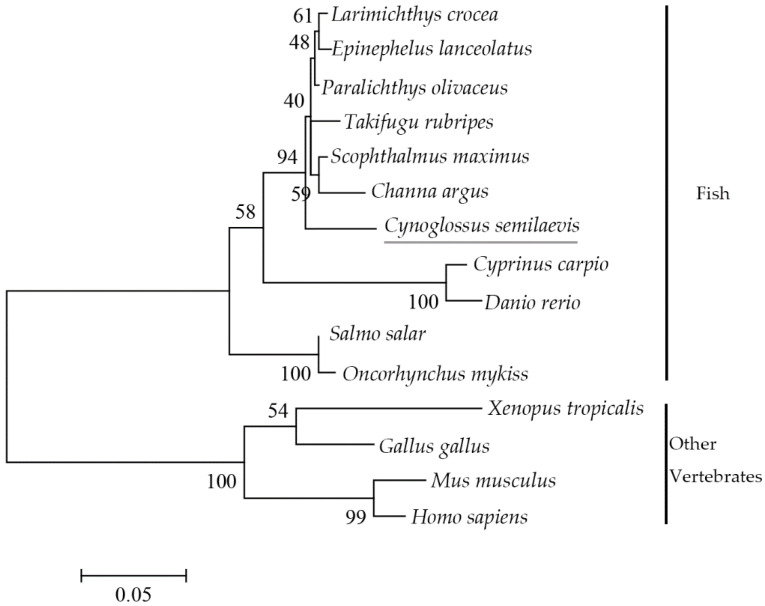
Phylogenetic tree of Cyp26b1 in Chinese tongue sole and other species based on the ML method. Bootstrap values were based on 1000 resampling replicates.

The GenBank accession numbers for Cyp26b1 from the Chinese tongue sole and 14 additional vertebrates are listed in Table 3. As shown in Figure 2, the homology analysis showed that Cyp26b1 from the Chinese tongue sole shared high identities (86.72–96.48%) to Cyp26b1 sequences in other teleost fish, while only 75.78% to humans and 75.98% to mice. The phylogenetic tree showed that *cyp26b1* genes were significantly clustered into two categories, where Chinese tongue sole and other fish formed one clade; the other vertebrates were grouped together (Figure 3).

### 3.3. The Spatiotemporal Expression of cyp26b1

**Figure 4:**
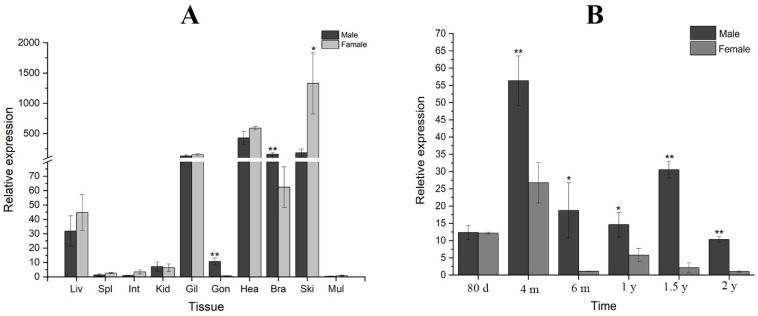
Relative expression of *cyp26b1* in Chinese tongue sole tissues. (**A**), relative expression of *cyp26b1* in different tissues. (**B**), relative expression of *cyp26b1* in various development stages. Values are indicated as means ± SEM (N = 3). ‘*’ refers to statistically significant differences (*p* < 0.05), and ‘**’ means very significant differences (*p* < 0.01).

The gene *cyp26b1* mRNA was identified in a variety of tissues in one-and-a-half-year-old Chinese tongue sole. It had a remarkably increased level of expression and no sexual dimorphism in the liver, gills, or heart (Figure 4A). Interestingly, *cyp26b1* mRNA showed significantly higher expression in the testis than in the ovaries (Figure 4A). There was no variation that was statistically meaningful between the ovaries and the testis at 80 dph in the Chinese tongue sole (Figure 4B). The expression of *cyp26b1* showed sexual dimorphism after 4 mph and higher level of expression in the testis than in the ovaries (Figure 4B). There was a tendency for the expression to decrease in the 6 mph and 1 yph testis (Figure 4B). Then, the expression is increased in the 1.5 yph testis and decreased in the 2 yph testis (Figure 4B). In contrast, the expression of *cyp26b1* was lowest in the 6 mph ovaries, and increased in the 1 yph ovaries, followed by decreases in the 1.5 yph and 2 yph ovaries (Figure 4B).

### 3.4. Cellular Localization of cyp26b1

**Figure 5:**
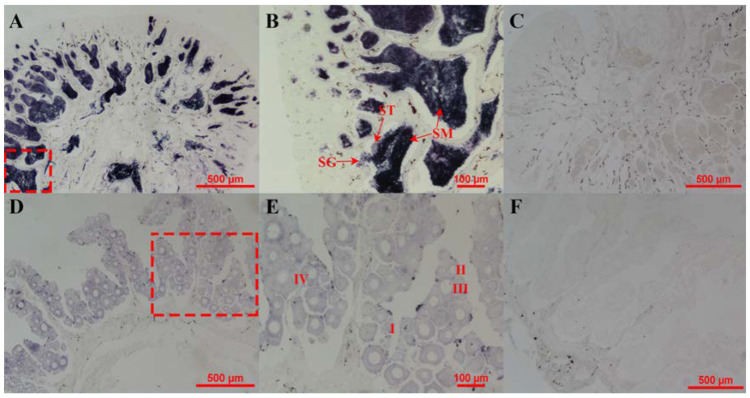
Cellular regionalization of *cyp26b1* mRNA in 2 yph gonads of Chinese tongue sole. In situ hybridization of gonads by means of antisense (**A**,**B**,**D**,**E**) and sense (**C**,**F**) probes of *cyp26b1* mRNA performed in Chinese tongue sole. (**B**) High magnification of the red-framed space in (**A**). (**E**) a high magnification of the red-framed space in (**D**). SG: spermatogonia; ST: spermatid; SM: sperm; I: Stage I oocytes; II: Stage II oocytes; III: Stage III oocytes; IV: Stage IV oocytes. Scale bar is shown in the figures.

The expression of the *cyp26b1* mRNA in the gonads was detected through in situ hybridization. The *cyp26b1* signals located mainly in spermatogonia, spermatids, and sperm were found to be highly intense, as revealed from the ISH results (Figure 5A,B). Further, in the ovaries, the signals were also spotted (Figure 5D,E).

## 4. Discussion

Cytochrome P450 enzymes are a superfamily of heme-containing monooxygenase enzymes that catalyze many oxidative reactions. In the RA-catabolizing reaction, the protein Cyp26b1, acting as the cytochrome P450 hydroxylase, could metabolize RA [25]. In mice, the gene *Cyp26b1* is expressed in fetal gonads and considered a key enzyme in RA degradation in the gonads [26]. In Japanese flounders, high temperature induces the expression of *cyp26b1* in the XX gonads and lead to the formation of XX masculinizational gonads [6]. In this study, the gene *cyp26b1* from the Chinese tongue sole has a conserved region that encodes a 441-amino acid p450 domain. This gene has the highest homology with the *cyp26b1* from other fish, so it was confirmed as the *cyp26b1* gene of the p450 family. The ORF of Chinese tongue sole *cyp26b1* was 1536 bp in length and encoded a 512 amino acid protein. However, the predicted protein of the *cyp26b1* gene from the Salmo salar and Oncorhynchus mykiss has 518 amino acids. The homology analysis shows that the similarity of the *cyp26b1* gene of the Chinese tongue sole with humans and mice was lower than that for other fish. This indicates that *cyp26b1* still has certain differences among species with different evolutionary positions.

In mammals, the gene *Cyp26b1* destabilizes ovary development; supporting testis development is an important component in the downstream of Sry and Sox9 located in ovary-antagonizing and testis-determining pathways [5,7,27]. During the sex variation of the XX gonads in the Japanese flounder, a high water temperature and cortisol induced *cyp26b1* mRNA expression and delayed the beginning of meiosis in GCs [6]. It was observed that the *cyp26b1* gene indicates a significant dimorphism in the gonads between the males and the females in the 1.5 yph Chinese tongue soles. The result of a different expression in the gonads with a higher expression in the testis is consistent with the expression pattern of *Cyp26b1* in mice [7,9,27]. For the development of the testis to move on normally, using the RA-degrading enzyme Cyp26b1, the endogenous RA needs to be cleared actively from the testicular tissue [12]. As a result of the genetic ablation of Cyp26b1 in mice, the ovotestis is formed, and the transcriptional procedures are interrupted by the presence of the ectopic RA. In turn, the *Mullerian-inhibiting factor* (*Amh*) and steroid hormones are produced in the XY gonads with downstream influences regarding secondary sexual development [27,28]. The in situ hybridization method was performed to detect the signals of *cyp26b1* in the gonads. The *cyp26b1* mRNA displayed a strong hybridization signal in the testis and a light hybridization signal in the ovaries, which was consistent with the results of the qPCR. In the testis, the strong hybridization signal was located in the spermatogonia, spermatid, and sperm. This suggests that *cyp26b1* may play a role in spermatogenesis. RA can elicit GCs to participate in meiosis in embryonic ovaries by down-regulating *cyp26b1* expression. In the male-specific upregulation of *cyp26b1* expression, the deprivation of the RA is caused by protecting the GCs from trying to enter meiosis in the emerging testes, as observed in the gonadogenesis of mice [11,12,29]. During the early stages of the Chinese tongue sole’s life, the GCs in the early testis and ovaries both forego meiosis. There is no significant difference in the *cyp26b1* expression of gonads. In 4 mph gonads, there was a significantly increased expression of *cyp26b1*. We speculate that it metabolized RA, resulting in suppressing meiosis and the growth of the gonads. Combining the above results, it can be surmised that *cyp26b1* could play a considerable role in the gonadal development and sex differentiation of the Chinese tongue sole.

Unlike the case of the Japanese flounder [6], the *cyp26b1* mRNA of Chinese tongue sole was not only expressed in the gonads and liver; rather, it was also highly expressed in the gills, heart, brain, and skin. The *cyp26b1* expression patterns of these tissues were even higher than the gonadal ones. In mice, a distinct skin-barrier homeostatic network was found to operate through *Cyp26b1* expressed highly in the fibroblasts of skin, and its suppression led to an increase in P2 × 7 expression in mast cells [30]. The *cyp26b1* mRNA was intensely articulated in the liver of the Japanese flounder and rats [6,31]. *Cyp26b1* also displayed other functions such as lymphatic vascular development, limb outgrowth, and morphogenetic growth [25,32,33]. It was believed that it plays a substantial role in the immune regulation of the Chinese tongue sole; nevertheless, this would require further experimental authentication.

## 5. Conclusions

In summary, we obtained the CDS of the *cyp26b1* gene in the Chinese tongue sole. The *cyp26b1* gene was primarily expressed in the gonads and other tissues. The expressed level of mRNA indicated that *cyp26b1* was mainly expressed in the testis. The ISH results revealed that *cyp26b1* was mostly detected in the testicular GCs. Hence, *cyp26b1* might impact the sex differentiation and gonadal growth in the Chinese tongue sole. Nonetheless, further experiments are needed to test the other functions.

## Figures and Tables

**Table 1:** Anesthetic doses for different fish ages and weights (dph: day post-hatching; mph: month post-hatching; yph: year post-hatching).

Age	Average Weight (g)		Anesthesia Dose (mg/L)	
	Male	Female	Male	Female
80 dph	1.24	1.26	10	10
4 mph	2.57	2.79	10	10
6 mph	17.38	23.80	60	60
1 yph	89.30	390.60	60	180
1.5 yph	195.00	1050.00	60	180
2 yph	300.40	1860.00	180	180

**Table 2:** Primer sequences used in this study.

Primer Name	Sequence (5′-3′)	Application	Product Size
Cyp26b1-F	ATGATGTTCGACACCTTTGACCTGG	Cloning the ORF	1536 bp
Cyp26b1-R	TCAGACGGTTGCTCCCAGAAGCTC		
q-Cyp26b1-F	AGTACCTGTGGTCCATCCTGTTGA	Real-time PCR	87 bp
q-Cyp26b1- R	TCTGACTTAGCCATGATCTCGTTCTG		
β-actin-F	GCTGTGCTGTCCCTGTA		150 bp
β-actin-R	GAGTAGCCACGCTCTGTC		
Cy-ISH-F	GGCTGCTGCAGGGTTCAA	in situ hybridization	443 bp
Cy-ISH-R	GGAGAACAGATGTTTCATCTCGTCT		
CS-sex-F	GAGGCCGACAGGATCGTAC	Sex genotype	206 bp/218 bp
CS-sex-R	TACGACGTACTCCGGTGGTTTT		

**Table 3:** Table list of species used in multiple alignment.

No	Species Name	GenBank Accession Number for Cyp26b1 Protein	GenBank Accession Number for *cyp26b1* Gene	Source
1	*Cynoglossus semilaevis*	XP_008322472.1	XM_008324250.3	Obtained in this study
2	*Paralichthys olivaceus*	XP_019951364.1	XM_020095805.1	GenBank
3	*Scophthalmus maximus*	XP_035462952.1	XM_035607059.2	
4	*Takifugu rubripes*	XP_003966921.1	XM_003966872.3	
5	*Larimichthys crocea*	XP_010742602.1	XM_010744300.3	
6	*Epinephelus lanceolatus*	XP_033465922.1	XM_033610031.1	
7	*Salmo salar*	XP_045557602.1	XM_045701646.1	
8	*Oncorhynchus mykiss*	XP_036843080.1	XM_036987185.1	
9	*Channa argus*	KAF3697672.1	CM015724.1	
10	*Cyprinus carpio*	XP_042583369.1	XM_042727435.1	
11	*Danio rerio*	NP_997831.1	NM_212666.1	
12	*Xenopus tropicalis*	NP_001072655.1	NM_001079187.2	
13	*Gallus gallus*	XP_015141554.1	XM_015286068.4	
14	*Mus musculus*	AAN08613.1	AY134662.1	
15	*Homo sapiens*	NP_063938.1	NM_019885.4	

## Data Availability

The data presented in this study are available in this article.
